# Correlation between human papillomavirus viral load and cervical lesions classification: A review of current research

**DOI:** 10.3389/fmed.2023.1111269

**Published:** 2023-02-21

**Authors:** Yilu Zhou, Xiaoyu Shi, Jiaxin Liu, Lina Zhang

**Affiliations:** Center for Diagnosis and Treatment of Cervical Diseases, Changzhou Maternity and Child Health Care Hospital, Changzhou Medical Center, Nanjing Medical University, Changzhou, China

**Keywords:** human papillomavirus, viral load, HPV genotyping, multiple infections, cervical lesions

## Abstract

Cervical cancer is the fourth largest malignant tumor among women in the world. Human papillomavirus (HPV) infection can lead to cervical intraepithelial neoplasia (CIN) and cervical cancer. Active papillomavirus infection occurs when the infected basal cells replicate and fill a certain area. Persistent HPV infection can lead to squamous intraepithelial lesions, which are divided into CIN1, CIN2, and CIN3 according to how much epithelium is impacted. Different types of HPV have different possibilities of causing cervical cancer, and high-risk HPV is the main cause of cervical cancer. Research showed that viral load may be an indicator of the progression of cervical precancerous lesions, but this association does not seem to be universal. This article aims to summarize different genotypes, multiple infections, especially viral load, in cervical precancerous lesions, to guide early intervention.

## 1. Introduction

Cervical cancer is a leading cause of mortality among women. In 2020, an estimated 604,000 women were diagnosed with cervical cancer worldwide and about 342,000 women died from the disease (1). According to epidemiological research statistics, in the United States, 75% of people aged 15–50 are infected with human papillomavirus (HPV) in their lifetime, of which 60% are only temporary infections, 10% are persistent infections (the habitual targets of the HPV), 4% have slight cytological changes, and only 1% have clinical cytological damage. Persistent infection with about 15 types of hrHPV is the main risk factor for cervical cancer, of which HPV16 and HPV18 infections account for about 70% of the total cases (2). In the summary analysis of 11 case–control studies (3), HPV 16, 18, 45, 31, 33, 52, 58, and 35 accounted for 95% of HPV DNA-positive squamous cell carcinoma.

Two decades ago, hrHPV testing was proposed as a potential alternative to repeated cytology or immediate colposcopy for the triage of women with Atypical Squamous Cells of Undetermined Significance (ASCUS) cytology (4). In the last few years, the superiority of hrHPV testing compared to cytology to detect high-grade lesions has been demonstrated (5). However, for young women aged 21–24, the specificity of the HPV mRNA test in defining CIN2 lesions in women with ASCUS or Low-grade Squamous Intraepithelial Lesion (LSIL) is much higher than hrHPV DNA test (6). Recently, a Cochrane review (7) pointed out that relevant triage strategies are needed to manage hrHPV-positive women. Biomarkers have been assessed to manage hrHPV-positive women that include HPV genotyping, p16/Ki67 dual-staining, or the methylation status of HPV and some human genes (8–10). According to recent longitudinal studies, hrHPV viral loads can affect cervical diseases to varying degrees. This article summarizes the research progress on the correlation between hrHPV viral load, HPV genotyping, and cervical lesions and provides guidance for the screening of cervical cancer.

## 2. Human papillomavirus genome

**Figure 1:**
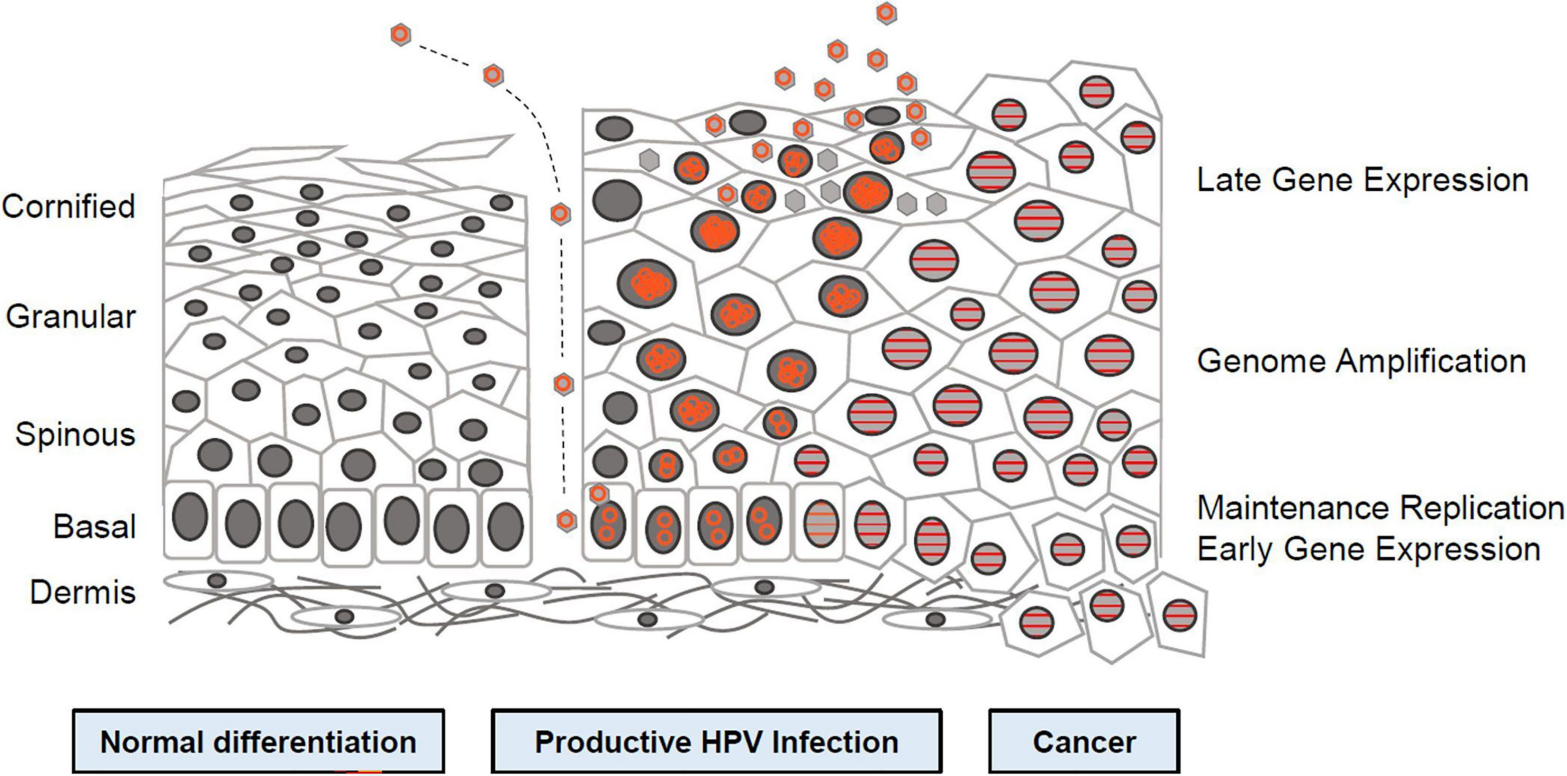
Human papillomavirus (HPV) life cycle and cancer (12). Cartoon depicting normal stratified cervical epithelium (left), HPV infected epithelium (center), and HPV induced cancer (right). Epithelial layers are indicated on the far left and HPV life cycle stages are indicated on the far right. Episomal genomes are shown as orange circles and integrated genomes shown as orange stripes. Left: Normal keratinocyte differentiation. Basal cells divide and daughter cells migrate upward, beginning the differentiation program. As differentiation proceeds, cells exit the cell cycle. Fully keratinized squames slough off from the apical surface. Middle: Productive HPV Infection: HPV virions gain access to basal cells *via* microwounds. The viral genomes migrate to the nucleus, where they are maintained at approximately100 copies/cell. As daughter cells begin differentiation, viral genomes are amplified. Cell nuclei are retained and chromatin is activated to support viral DNA replication. Right: Cancer. Viral genomes often integrate into the host genome and E6/E7 expression is increased, leading to enhanced proliferation and accumulation of cellular mutations. Cellular differentiation is lost and cancerous cells invade into the dermal layer along with neighboring tissues (12).

Human papillomavirus is a DNA virus in the papillomavirus subgroup. The shell consists of 72 5-polymers, 20 polyhedra, no envelope, 45–55 nm in diameter, and 5 × 106 Da. The HPV genome is an annular double-stranded DNA molecule with about 7,800–7,900 base pairs (bp), whose DNA composition accounts for about 12% of the mass of the virus (11). The complete genome can be divided into 3 coding regions (Figure 1): (1) early region (open reading box): including six genes in total, including E1, E2, E3, E4, E5, and E6, with a total length of about 4,500 bp, which can participate in the replication, transcription, and cell transformation of viral genes; (2) advanced regions (late stage) Coding area: contains a total of 2 genes, L1 and L2, of which L1 is the main capsid protein and L2 is the secondary capsid protein, which can be self-assembled into viral-like particles to induce the body’s immune response and promote the production of protective antibodies. It belongs to the late expression of viral replication; (3) Upstream regulatory area (long control) control area, non-coding area: Located between the L1 gene and the E6 gene, it contains multiple binding sites and can participate in the regulation of virus replication and transcription (12).

## 3. Human papillomavirus genotyping

Of the approximately 30 types of HPV that infect the anogenital tract, 15 types of HPV, classified as “high-risk” types (HPV types 16, 18, 31, 33, 35, 39, 45, 51, 52, 56, 58, 59, 68, 73, and 82), are associated with high-grade lesions and invasive cervical cancer (13). On the other hand, 11 different HPV types, classified as “low-risk” HPV types (HPV types 6, 11, 40, 42, 43, 44, 54, 61, 70, and 81), are mainly associated with genital warts and benign cervical lesions (14). In the list of type 1 carcinogens published by the International Agency for Research on Cancer of the World Health Organization, there are types 16, 18, 31, 33, 35, 39, 45, 51, 52, 56, 58, and 59 of high-risk HPV (11).

### 3.1. Single HPV infection

The global prevalence of five top hrHPV types among women (15) is reported to be HPV 16: 55.4 (95% CI; 55.0–55.8), HPV 18: 14.6 (95% CI; 14.3–14.9), HPV 45: 4.8 (95% CI; 4.6–5.0), HPV 33: 4.2 (95% CI; 4.1–4.4), HPV 58: 3.8 (95% CI; 3.7–4.0), and HPV 31: 3.5 (95% CI; 3.4–3.7). A global study on HPV genotypes (16), published in the Lancet in 2010, found that genotypes HPV 16, HPV 18, HPV 31, HPV 33, HPV 35, HPV 45, HPV 52, and HPV 58 are easily capable of causing moisturizing cervical cancer. As previously stated, HPV 16/18 is the primary virus of cervical intraepithelial neoplasia (CIN). However, Ma L (17), on the other hand, found that CIN2+ accounted for 31.02% of 648 HPV-positive histopathological data, with HPV16 having the highest infection rate and HPV18 having only 3.75%, but HPV18 can play an important role in severe cervical lesions, with CIN3 and cervical adenocarcinoma being closely related to HPV18 single infection. A recent systematic synthesis (18) showed that individual HPV genotypes carry distinct risk values for high-grade cervical disease. HPV16 consistently carries the highest risk for CIN 3 or worse, HPV31, 18, and 33 carry intermediate-high CIN 3 or worse risk. Beyond HPV 16, 31, 18, and 33, HPV 52, 58, and 45 carry moderate risks, with 35, 39, 51, 56, 59, 66, and 68 consistently having the lowest CIN 3 or worse risks.

### 3.2. Multiple HPV infections

Women may be infected with multiple HPV infections with different genotypes throughout their lives (3). It is reported that among HPV-positive women, the prevalence rate of multiple HPV infections is between 18.5 and 46% (19–21). A variety of HR-HPV types infect synergistically in the occurrence of cervical cancer, according to prospective research (22). Fife et al. (23) reported that infection with multiple HR-HPV types tends to increase the severity of cervical diseases. However, other reports provided controversial conclusions. Muñoz et al. (3) found that there is no significant difference in the risk of cervical cancer among women with multiple and single HPV infections. Herrero et al. (24) have shown that multiple infections may be related to the persistence of HPV and increase the duration of infection and the risk of cervical disease, but some studies (25–27) still showed no impact. Recently, Iacobone et al. (28) confirmed that multiple HPV infections are significantly associated with reduced CIN2+ risk, while cervical cancer and changes in precancerous diseases may occur in single infections. Another hrHPV test through Cobas4800 showed that the risk of HPV16 co-infection with other types of CIN3 seems to be lower than that of a single HPV16 infection (29). A study (30) from Beijing, China, also indicated that the incidence of CIN2+ in patients with a single HPV 16 infection (62.2%) is higher than that of patients mixed with other HPV genotypes (52.4%). At the same time, the incidence of CIN2+ in patients infected with HPV 16 may be higher than that of patients with a single HPV 52 and other genotypes. Recently, a study published by Song et al. (31) suggested that co-infection with lower-grade HPV types has little impact on the CIN2 + risk associated with a single hrHPV infection, which confirms the above conclusion.

## 4. Human papillomavirus viral load

### 4.1. hrHPV viral load and cervical lesions

Most scholars (32–41) believe that there is a clear correlation between HPV viral load and the degree of cervical lesion, that is, as the viral load increases, the risk of cervical lesions increases. A large Chinese retrospective study (34), compared the viral load of ≤ CIN1 and CIN2 + patients in eight high-risk HPV genotypes (HPV16/18/31/33/45/52/58/82). The results showed that statistical significance was only found in HPV16 genotypes; there was no such difference in the other seven genotypes. Zhao et al. (36) conducted a 15-year prospective cohort study in China and found a significant correlation between the change in HPV viral load over time and the probability of CIN2+ in patients. Women with an increased viral load (15.3%) had a 38-fold higher risk of CIN2+ than HPV-negative women (0.4%). We summarized some longitudinal studies (Table 1) published in the past 5 years and found that the HPV viral load is indeed related to the CIN level of cervical lesions, but this law is not universal. It needs to be reflected in a specific genotype. For example, in the cohort of French women (42), only the viral load of HPV16 can predict CIN2+, and this association has not been found in HPV18 and other genotypes. This rule has also been found in the Chinese female cohort (34). More interestingly, a cohort study from Canada (33) showed that the HPV 16/18/31 viral load is related to higher levels of cervical lesions. In the Mexican women’s cohort (35), we found that women in LSIL and HSIL have a higher HPV 16 viral load. In contrast, Del Río-Ospina L et al. (44) study in Colombia has documented that a higher level of cervical lesions in women corresponds to a lower HPV 16 viral load. Wang W et al. (39) have shown that the HPV16 viral load can gradually increase with the development of the lesion, and there is no obvious correlation between the HPV18 viral load and histopathology. At the same time, the viral load of subtypes close to HPV16 (HPV52, HPV58, etc.) can increase with the development of the disease, while the viral load of subtypes close to HPV18 (HPV45, HPV59, etc.) does not change significantly. Unlike the general description of viral load two decades ago, the description of viral loads of different HPV genotypes in recent years has helped us better understand the relationship between viral load and CIN classification.

**Table 1:** Longitudinal studies.

Author	Year	Country	Participants	Findings
Xiang (34)	2022	China	17,235	The most prevalent hrHPV genotype for study patients who had ASC-US cervical cytology results were HPV52 (16%), HPV16 (11.3%), HPV58 (10.2%), and HPV53 (8.4%). The least prevalent hrHPV genotypes were HPV26, HPV82, HPV73 and HPV45, each with a prevalence < 1.5%.
				Only the viral load difference between < CIN1 and CIN2+ groups (*p* = 0.001) was found in HPV16, and there was no such difference in the other seven genotypes.
Liu (32)	2021	China	256	Higher high-risk HPV viral load in cervical lesions is related to higher risk of high-level cervical lesions.
Baumann (42)	2021	French	885	HPV16 DNA load may independently predict the development of CIN2 + .
				On the contrary, compared with other hrHPVs, contracting HPV 18 does not increase the risk of high-level cervical disease development.
Oyervides (35)	2020	Mexican	294	The predominant HPV type was 16 (33.7%), followed by 18 (25.3%) and 39 (22.5%).
				We found associations between HPV 18, 51, and 56 and high viral loads (*p*-values of 0.012, 0.034, and 0.005, respectively).
				Significant difference was also not found when we analyzed HPV 18 and HPV52.
Zhao (36)	2019	China	1,479	Individuals with medium/high virus loads, especially those whose HPV load remains stable, as well as individuals with increased HPV loads, are at greater risk of high-level lesions in the future and may be used as triage indicators for HPV-positive women.
Talía (33)	2019	Canada	1,611	Viral load varied by HPV type and by diagnosis. The geometric mean viral load was highest for HPV16 and lowest for HPV45.
				Higher HPV16/18/31 viral load was associated with a higher likelihood of being diagnosed with CIN and cancer.
Long (43)	2017	America	2,902	The viral load of subtypes close to HPV16 can increase with the development of the disease.
				The viral load of other types appeared slightly lower among women with HSIL compared to those with LSIL.
Del L (44)	2015	Colombian	180	The increased viral load of HPV-18 and HPV-33 is directly proportional to the degree of cervical lesions.
				High frequency of cervical lesions in women with low HPV-16 load.

However, some experts (45–47) do not think that HPV viral load is associated with the degree of cervical lesions (48). They said that CIN1 was in the acute stage of HPV infection, and HPV’s self-replication ability was significantly more prominent in other stages. When CIN1 developed into CIN2-3, there was a significant downward trend in HPV viral load because HPV’s self-replication ability was relatively stable. When it developed from CIN2-3 to cervical cancer stage I again, the HPV gene was integrated into the DNA of the host cell and had the ability to transform cells, causing the cervical lesion to gradually transform into cervical *in situ* cancer and even infiltrating cancer (48, 49). From this perspective, the viral load may underestimate the severity of the disease, thus delaying treatment.

When we reviewed these studies, we also found that the viral load is also related to age. Compared with women under the age of 30, the relationship between viral load and cervical lesions in women older than 30 years old is stronger (33).

### 4.2. Viral load as a triage biomarker

At present, many countries around the world use HPV testing for primary cervical cancer screening. Clinical trials (50) showed that HPV detection and screening of high-level lesions are more sensitive than cytological testing. The randomized clinical trial published in JAMA Oncology (51) indicated that genotyping for hrHPV with cytology triage significantly reduced the colposcopy referral rate compared with cytology for urban women. However, many HPV-positive women have no potential cervical lesions. To avoid overburdening colposcopy services and reduce the harm caused by over-referral, HPV-positive women must undergo a second test. Whether the HPV viral load can be used as a biomarker for triage is a question worth discussing at present. Luo et al.’s (52) research used the viral load as a triage indicator for cervical cancer screening. It is recommended that colposcopy be performed immediately when the viral load is > 10 RLU/CO, and cytological testing should be carried out at > 1 RLU/CO or < 10 RLU/CO to optimize sensitivity, specificity, and the referral rate. This proved that the viral load can be used as a triage indicator. However, The HPV viral load can only predict the risk of cervical lesions under specific HPV typing. This may not apply to all HPV-positive women.

In December 2021, the World Health Organization issued guidelines for the screening and treatment of cervical precancerous lesions, proposing the use of mRNA testing for cervical cancer screening. Even if the viral load cannot be used as a method for secondary triage, it can be used as an indicator of virus replication to predict condition tracking and subsequent treatment.

## 5. Discussion

Due to the close relationship between HPV and cervical cancer, cervical cancer becomes a unique type of cancers. Its etiology is clear, and early prevention and diagnosis can achieve complete eradication. In May 2018, the Director-General of WHO called on countries to take action to jointly achieve the global goal of eliminating cervical cancer. Vaccinating adolescents against HPV is now the primary cervical cancer prevention strategy (53). However, HPV vaccination is not recommended instead of cervical cancer screening (54), although the vaccine can greatly reduce the risk of cervical cancer (55). Regardless of the relationship between HPV viral load and cervical lesions, viral load must be combined with other methods to maximize sensitivity and specificity in cervical cancer screening. Therefore, we need to further study the many factors that may influence the occurrence and development of cervical lesions, to achieve the combined application of multiple methodological detections in the early screening of cervical cancer, which is conducive to the early detection, early diagnosis, and early treatment of cervical cancer, so as to achieve the elimination of cervical cancer by the World Health Organization by 2030.

## Author contributions

LZ and YZ conceived the review study. YZ drafted the manuscript. All authors contributed to the article and approved the submitted version.

## Funding

This study was funded by grants from The Maternal and Child Health Research Project in Jiangsu Province (F202166) and The Changzhou Health Commission Guidance Project (WZ202217).

## Conflict of interest

The authors declare that the research was conducted in the absence of any commercial or financial relationships that could be construed as a potential conflict of interest.

## Publisher’s note

All claims expressed in this article are solely those of the authors and do not necessarily represent those of their affiliated organizations, or those of the publisher, the editors and the reviewers. Any product that may be evaluated in this article, or claim that may be made by its manufacturer, is not guaranteed or endorsed by the publisher.
